# Impact of ICT diffusion and opportunity entrepreneurship on environmental sustainability in Saudi Arabia

**DOI:** 10.1016/j.heliyon.2024.e39009

**Published:** 2024-10-05

**Authors:** Faisal Alfehaid, Anis Omri, Ahmad Altwaijri

**Affiliations:** Department of Business Administration, College of Business and Economics, Qassim University, Qassim, Saudi Arabia

**Keywords:** ICT, Opportunity entrepreneurship, Environmental sustainability, Non-linearity, Complementarity

## Abstract

The need to prioritize sustainable development is expanding in light of the world's environmental concerns. To address these concerns, entrepreneurship is essential as a catalyst for inventions, economic expansion, and social change. Entrepreneurship activities have direct and indirect effects in the face of environmental risks and uncertainties. This study addresses gaps in understanding entrepreneurship's non-linear and complementarity effects, particularly opportunity-driven entrepreneurship and information and communication technologies (ICT), on the quality of the environment in Saudi Arabia. The dynamic ordinary least squares (DOLS) method is used to estimate long-run relationships. Saudi Arabia will be the perfect context for sustainability because the country has prioritized sustainability through its Vision 2030, and the Saudi government has substantially supported entrepreneurship. The main contribution of this paper to the existing literature is evident in its examination of the quadratic relationships between both opportunity-driven entrepreneurship and ICT diffusion, including ICT access, use, and skills, on environmental quality. In addition, the study delves into the ICT diffusion's modulating effects on the nexus of opportunity entrepreneurship with environmental quality, providing insights into how these factors can effectively improve environmental quality. The findings show that opportunity entrepreneurship and ICT diffusion initially deteriorate environmental quality before leading to improvements as their levels mature in the economy. Moreover, interactions between ICT proxies and opportunity entrepreneurship yield mixed effects, with negative net effects on CO_2_ emissions and ecological footprint countered by positive net effects on the environmental performance index. These findings highlight the dual role of ICT diffusion as a contributor to environmental challenges and a potential solution, depending on the level of its diffusion and interaction with entrepreneurial activities. Therefore, policymakers should create plans that encourage and direct business activity toward more environmentally friendly methods. They should also consider the short- and long-term effects of growing digital technologies on environmental sustainability and how they might revolutionize how entrepreneurship and sustainability goals are aligned.

## 1 Introduction

Given the global environmental challenges, there is a growing necessity to prioritize sustainable development. Within this context, the concept of environmental sustainability, which emphasizes responsible management of our natural resources and the mitigation of ecological degradation, has captured the attention of policymakers, scholars, and practitioners worldwide [1]. Guided by the principles outlined in “The Future We Want” document by the United Nations, the international community has committed to advancing sustainable development goals (SDGs), which cover a wide range of goals, such as alleviating poverty, promoting gender equality, taking action on climate change, and encouraging sustainable consumption and production.

Entrepreneurship plays a crucial role as a catalyst for innovations, economic growth, and social change in achieving these goals [2]. Specifically, opportunity entrepreneurs, who have a keen eye for identifying and seizing market opportunities to create value through innovative solutions [3], play a pivotal role in driving positive environmental outcomes [4]. By harnessing their creativity, ingenuity, and market insights, they develop ventures that address environmental issues while producing economic value. These entrepreneurs focus on green energy, eco-friendly technologies, and sustainable agriculture, contributing to the advancement of environmental sustainability objectives outlined in the SDGs. Their initiatives not only promote environmental stewardship but also have positive ripple effects such as job creation, community empowerment, and economic resilience in the face of environmental risks and uncertainties (Altwaijri et al., 2024). Some scholars, including Omri and Afi [5], He et al. [6], and Altwaijri et al. (2024), have attributed this positive impact of entrepreneurial activity to the presence of certain conditions, such as strong institutions, environmental innovation, ICT diffusion, and education. On the other hand, entrepreneurship, while often associated with economic growth and innovation, can harm environmental quality, particularly in developing nations. Ben Youssef et al. (2018) and Dhahri et al. [4] highlighted in their studies that entrepreneurship, whether driven by opportunity or necessity, can negatively affect the environment. The negative impact of entrepreneurship on environmental quality in developing economies underscores the urgent need for implementing sustainable business practices, enforcing environmental regulations, promoting eco-friendly technologies, and fostering awareness among entrepreneurs about the necessity of environmental conservation for long-term economic and social well-being [6]. Another strand of studies has recently focused on the sustainability effect of ICT diffusion, highlighting negative and positive effects [7]. ICT can potentially influence environmental sustainability in two ways (Higon et al., 2017). On one hand, ICT's usage, production, and disposal phases are associated with negative environmental outcomes. For example, the electricity consumption of ICT systems can lead to increased carbon emissions. In this sense, Røpke and Christensen [8] argued that the environmental footprint of ICT extends beyond energy usage to encompass manufacturing processes and the maintenance of infrastructure, such as server farms and data centers, which contribute significantly to environmental degradation. In contrast, ICT holds promise for environmental conservation, providing ways to decrease emissions by improving transport systems, smarter cities, electricity grids, and industry processes. This perspective, often termed green ICTs, views ICT as a pivotal tool in addressing various environmental challenges, including climate change, prompting optimism among researchers regarding its potential benefits (Higon et al., 2017; [9,10]). Although most prior research has concentrated on examining the linear and conditional impacts of both opportunity entrepreneurship and ICT on the quality of the environment, little work has been done in exploring potential non-linear associations. Current research has yet to thoroughly investigate the potential modulating influence of ICT diffusion on the nexus between opportunity entrepreneurship and environmental improvement. This gap in knowledge highlights the need for further exploration. In essence, when considering the role of ICT in fostering opportunity entrepreneurship, it becomes evident that it is crucial in advancing environmental sustainability. By leveraging ICT, opportunity entrepreneurs can enhance their capacity for innovation [11], improve operational efficiency [12], and adopt sustainable practices within their supply chains [13].

To fill these gaps, this article aims to demonstrate the non-linearity and complementarity effects of entrepreneurship, specifically opportunity-driven entrepreneurship and ICT, on environmental quality in Saudi Arabia. Saudi Arabia has chosen for several compelling reasons. First, Saudi Arabia has set sustainability as a top priority through its Vision 2030, which aims to achieve sustainability objectives and accelerate the shift to renewable energy sources to reach net-zero emissions by 2060 (Saudi Green Initiative). This strategic vision has spurred the launch of various initiatives and projects, such as The Line and NEOM, which directly and indirectly affect sustainable development efforts. Preliminary assessments of sustainability initiatives indicate that the propagation of Vision 2030 has increased awareness among the Saudi population about sustainable economic practices, environmental preservation, and social responsibility. One of the primary objectives of Vision 2030 is to strengthen the contribution of entrepreneurship to the local economy. According to the latest GEM (2022) report, many Saudi entrepreneurs and business owners prioritize social considerations in their business decisions. This includes aspects such as healthcare access, safety, educational opportunities, and overall quality of life. Moreover, there is a notable inclination among these entrepreneurs toward environmental concerns, including the conservation of green spaces, reduction of toxic emissions, and promotion of sustainable consumption patterns. Interestingly, these environmental concerns appear to be more pronounced among entrepreneurs than established business owners. Second, the Saudi government has taken significant measures to support entrepreneurship and its objectives. This is evidenced by the establishment of dedicated entities aimed at fostering entrepreneurship and the recent announcement of a 1.1 billion SAR fund to bolster entrepreneurship initiatives. Therefore, it is crucial to examine the effectiveness of entrepreneurship in advancing sustainability goals within the context of Saudi Arabia. Our findings can be generalized to GCC countries due to the similarity in terms of economic size and growth, demographic factors, geographical location, and cultural heritage. GCC countries are actively pursuing diversification strategies to diversify their economies towards a non-oil based including tourism, finance, and technology. In addition, GCC countries have a youthful population with a median age of 30 years. Moreover, the education system in GCC countries encourages innovation through many initiatives such as supported research and scholarship programs.

This article makes the following contributions: First, to examine their effects on improving the environment, ICT and entrepreneurship are considered sustainability components in the standard EKC model. The specific goal is to show how opportunity entrepreneurship and ICT diffusion initially deteriorate environmental quality, whereas environmental quality begins to improve later, once the levels of opportunity entrepreneurship and ICT diffusion have matured in the economy. Second, instead of merely investigating whether opportunity entrepreneurship and ICT diffusion contribute to environmental improvement, this article further delves into the mechanisms through which opportunity entrepreneurship can facilitate this objective. This examination specifically focuses on the moderating impacts of ICT diffusion, encompassing factors such as ICT access, usage, and skills as potential mechanisms that moderate the influence of opportunity entrepreneurship on environment quality. By examining these moderating effects, the article aims to better understand how opportunity entrepreneurship, in conjunction with ICT diffusion, can effectively contribute to environmental improvement. In terms of results, the study finds a positive association between opportunity entrepreneurship and environmental degradation while revealing a quadratic relationship suggesting that beyond a certain threshold, increased opportunity-driven entrepreneurs may improve environmental performance. Similarly, increased ICT diffusion levels negatively impact environmental quality, but beyond a certain threshold, further increases can lead to improved environmental conditions. Moreover, interactions between ICT proxies and opportunity entrepreneurship yield mixed effects, with negative net effects on CO_2_ emissions and ecological footprint countered by positive impacts on the environmental performance index. These findings show the dual role of ICT diffusion as a contributor to environmental challenges and a potential solution, depending on the diffusion level and its interaction with entrepreneurial activities.

The remaining sections of the article are organized as follows. The second section covers the related literature. The third Section describes the methodology and the used data. Section four presents and discusses the key findings. The last section offers a summary of the obtained results and recommendations for policymakers and practitioners.

## 2 Literature review

### 2.1 Entrepreneurship and environmental quality

At the global level, environmental challenges are becoming increasingly urgent. Entrepreneurs are acutely aware of the need for change to incorporate sustainable practices into their business models [4]. The 2030 Agenda for Sustainable Development urges societies to take decisive and transformative actions to steer the world toward a resilient and sustainable future. Entrepreneurship was therefore acknowledged as a key factor in driving this transformation ([14]; UN General Assembly, 2015; [4,15]). In this context, Perrini et al. [16] contend that entrepreneurs should concentrate on designing initiatives that balance the social, environmental, and economic results of their activities and entertain sustainable procedures in their pursuit of competitiveness and efficiency in the three dimensions of sustainability. Besides creating businesses to address many environmental and social concerns, entrepreneurship has also been a key tool for introducing sustainable services and goods [15]. Similarly, Xing et al. [17] argue that the entrepreneurship influence on environmental quality changes with innovation and creativity. Innovation lies at the heart of entrepreneurship's contribution to environmental sustainability. Entrepreneurs are at the forefront of developing cutting-edge technologies and business models that prioritize environmental conservation, including advancements in renewable energy, waste-reduction strategies, sustainable agriculture, and the promotion of circular economy principles. In addition, innovation in entrepreneurship activities can improve energy efficiency and reduce industrial emissions, which contributes to positive environmental outcomes.

There are diverse thoughts, approaches, and empirical studies available in previous literature, including social entrepreneurship, sustainable entrepreneurship, and ecopreneurship, that have analyzed the connection between sustainable development and entrepreneurship, with many research efforts concentrating on the environmental impacts of entrepreneurship. However, their results are mixed and inconclusive. The first group of studies found that entrepreneurship has a negative effect on environmental quality. For instance, using data from 69 countries, Omri [15] investigated the impact of entrepreneurial activity on the quality of the environment. It found that environmental damage caused by entrepreneurship in developed economies is lower than it is in developing economies. In the same spirit, Dhahri et al. [4] used data from 20 developing countries to reinvestigate the impact of opportunity and necessity entrepreneurship on sustainable development in its three dimensions. The authors demonstrated that both entrepreneurship types negatively influence the environmental dimension, and opportunity entrepreneurship has less of an effect on environmental degradation compared to necessity entrepreneurship.

Another strand of studies shows that entrepreneurship is a good vehicle to improve environmental quality. For instance, York and Venkataraman [18] showed that entrepreneurship improves environmental quality instead of degrading it. Similarly, He et al. (2020) found that environmental quality can be improved by entrepreneurship through improving agricultural practices, protecting the ecosystem, and reducing environmental deforestation. The authors concluded that by increasing the number of opportunities, entrepreneurs could be used as a solution to mitigate climate change and the degradation of the environment. Other scholars have attributed this positive impact to certain conditions. For example, Ben Youssef et al. (2018) similarly attributed this positive impact of entrepreneurial activity to the presence of good governance and green innovation. Lastly, the third strand of studies found an inverted U-shaped linkage between entrepreneurship and environmental degradation. For example, Omri [15] found that entrepreneurship in high-income economies at first damages the environment, but then recovers the environment after reaching an advanced level of entrepreneurship. Therefore, increased entrepreneurship does not always harm environmental quality. He et al. [6], for example, explored the influence of opportunity entrepreneurship on the quality of the environment from an institutional perspective. Their main findings show that entrepreneurship has a positive impact on improving environmental quality in the presence of strong institutions. The present study aligns with this last strand of literature and aims, therefore, to demonstrate how entrepreneurship (opportunity entrepreneurship) deteriorates environmental quality at the initial stage, whereas environmental quality later improves once opportunity entrepreneurs have matured in the economy. Hence, the first hypothesis is as follows.

Hypothesis 1 There is an inverted U-shaped relationship between opportunity entrepreneurship and environmental degradation in Saudi Arabia.

### 2.2 ICT and environmental sustainability

With the growing urgency to reduce carbon emissions and combat climate change, experts and scientists are turning to the effectiveness of ICT in improving energy production and efficiency [19]. The negative and positive environmental effects of ICT, which are broadly debated, can, therefore, have both negative and positive influences on the sustainable environment (Higon et al., 2017). ICT use, disposal, and production negatively contribute to the environment, particularly by increasing carbon emissions from the electricity required to operate them. Røpke and Christensen [8] argue that the environmental footprint of ICTs extends beyond direct electricity use to include energy consumed in the manufacturing of devices and the maintenance of infrastructures, such as server farms and data centers. The ICT sector is responsible for approximately 2 % of carbon emissions, as estimated by Mingay [20], leading to the concept of “green ICTs,” which aims to mitigate the environmental effects associated with ICTs [21]. In contrast, the ICT sector has beneficial effects on the environment, providing ways to decrease emissions through improving transport systems, smarter cities, electricity grids, and the processes of industry. This “green ICTs” perspective sees ICTs as a key tool for solving various environmental issues, and many studies express optimism about their role in mitigating climate change and other environmental problems (Higon et al., 2017).

Empirically, a growing number of research has recently focused on the influence of ICTs on sustainable environment in different regions and countries; however, their findings are inconclusive and contradictory. The first strand of research found a positive association between environmental degradation and ICT, particularly CO_2_ emissions. For instance, Lee and Brahmasrene [22] studied the influence of ICT on CO_2_ emissions in Asia, and they found evidence that ICT, particularly internet use, increases emissions. Using data from emerging countries, Danish et al. [23] investigated the effects of ICT and GDP on the quality of the environment; their findings also show that mobile phone subscriptions and internet use deteriorate the quality of the environment. However, another strand of studies found a positive effect of ICT on a sustainable environment. For example, Asongu et al. (2018) utilized the generalized method of moments (GMM) technique to empirically explore the effects of ICT on environmental sustainability using data from 44 sub-Saharan African nations between 2000 and 2016. Their results support the idea that ICs can help reduce carbon emissions from liquefied gasoline. Similarly, Tzeremes et al. (2022) used the cutting-edge GMM-PVAR method to determine the relationship between the quality of the environment, energy transition, and ICT for the BRICS countries. They discovered that ICT may be a key tool for energy transition and the resolution of environmental concerns. Their findings urge governments in the BRICS nations to support the development of ICT. Coroama et al. [24] argue that ICT contributes to environmental sustainability by enhancing energy efficiency as it induces a reduction in carbon emissions. This decrease is particularly attributed to the positive effects of using ICT applications, such as smart grids, intelligent transport systems, industrial processes, energy-saving gains, and smart buildings (Charfeddine and Kahia, 2021). A systematic study of 166 scientific articles by Charfeddine and Umlai (2023) examined 297 relationships between digitization/ICT and environmental quality. The findings demonstrate that while most studies found evidence that ICT and digitalization improve environmental sustainability, studies on the ‘group of” nations tended to find evidence against this association. Based on the study findings, it is evident that ICT has a substantial impact on reducing global CO_2_ emissions. Interestingly, these effects are particularly prominent in nations belonging to the OECD (Nejad and Shirazi, 2022). Finally, the third strand found an inverted U-shaped relationship between sustainable environment and ICT. For example, Higon et al. (2017) investigated the non-linear relationship between a sustainable environment and ICT from a global perspective, and they found a clear inverted U-shaped connection between both variables. The authors argue that numerous developed countries have reached the level of ICT development, resulting in diminishing environmental degradation as this development level increases. As a contribution to this last strand of research, the present study also aims to assess the non-linear relationship between a sustainable environment and ICT diffusion (use, access, and skills), i.e., environmental degradation is expected to rise as ICT diffusion increases, whereas, at a more advanced stage of ICT diffusion, environmental degradation may be mitigated. Therefore, the second hypothesis is as follows.

Hypothesis 2 There is an inverted U-shaped relationship between ICT diffusion and environmental degradation in Saudi Arabia.

### 2.3 Entrepreneurship, ICT, and environmental sustainability

Schreyer (2001) states that ICTs encompass computer hardware, software, and wired services. As the new decade dawns, these technologies have completely transformed a wide range of industries. It is impossible to halt the advancement of technology; instead, adapting and evolving with it is crucial. Gomes and Lopes [25] contend that ICT is an indispensable tool for entrepreneurs to maximize their resources and generate value. Consequently, the correlation between entrepreneurship and resource-based perspective theory has been a topic of great interest (Alvarez and Busenitz, 2001). Therefore, it is widely recognized that ICT plays a crucial role in enhancing productivity and driving economic growth [26]. According to the hypothesis proposed by Wade and Hulland [27], Rivard et al. [28], and Zhang and Li [29], the incorporation of ICT provides businesses with a vital and invaluable source of diverse resources, enabling them to gain a competitive edge in the information age. This dissemination of ICT has greatly simplified numerous business operations, laying the foundation for entrepreneurial development across various industries. First, by facilitating the identification and pursuit of new ideas and opportunities, such as expanding into new markets, establishing partnerships, and executing mergers [[29], [30], [31]], ICT empowers business owners to innovate continually. Additionally, the capacity of ICT to enhance communication and facilitate networking with suppliers is a crucial aspect of entrepreneurial success. Incorporating ICT into business operations provides numerous benefits, such as streamlining processes, reducing transaction costs, and facilitating the exchange of information [29]. For entrepreneurs, access to high-quality and abundant information through ICT is essential in gaining and sustaining a competitive advantage. In fact, as pointed out by Ghosh and Ghosh [32], entrepreneurs play a vital role in disseminating and acquiring knowledge, which is crucial for firms in transforming and integrating knowledge to gain a competitive advantage. This, in turn, is an integral aspect of a knowledge-based economy, as highlighted by Cuevas-Varga et al. [33]. According to Schaltegger and Wagner [34], ICT contributes to the emergence of sustainable business practices due to its facilitating efficiency improvements, reducing resources used in enterprises, and enhancing environmental performance. Such technologies provide the necessary instruments for companies to track and improve the efficiency of their operations. This results in significant energy and waste savings, making it possible to reach sustainability goals. ICT also provides entrepreneurs easy access to information, market trends, research, and best practices related to sustainability, enabling them to make informed decisions and develop innovative solutions that align with environmental and social objectives. It also offers possibilities for new practices that provide entrepreneurial opportunities and enable the development of new economic models [35]. Furthermore, a detailed analysis by Nambisan [36] focusing on digital entrepreneurship shows that ICT enables opportunity entrepreneurs to create and develop sustainable innovations, a feat that has never been seen previously. Digital platforms, cloud computing, and big data analytics give entrepreneurs the necessary instruments to quickly prototype, test, and improve green solutions. This significantly reduces the time and resources needed to bring sustainable innovations to the market.

In summary, it can be said that opportunity entrepreneurship, enabled by ICT, is a major factor in the advancement of environmental sustainability. Innovation, efficiency improvements, and sustainable supply chain management provide opportunities for entrepreneurs with the necessary tools to make important contributions to environmental sustainability. Hence, the third hypothesis is as follows.

Hypothesis 3 ICT diffusion modulates the effects of opportunity entrepreneurship on environmental quality.

## 3 Methodology and data

### 3.1 Model specifications and research method

Since incorporating sustainable development issues in Vision 2030, policymakers have searched for practicable policies and instruments designed to achieve environmental sustainability. In this context, entrepreneurship and ICT are important components in achieving this bold target. Their value is based on innovating and developing sustainable solutions for different sectors. Therefore, a theoretical model can be proposed which explores the dynamic interplay between opportunity entrepreneurship (OpE) and ICT diffusion as influential factors in promoting environmental sustainability (E):

(1) $$E_{t}=a_{0}^{+}{a_{1}OpE}_{t}+a_{2}{ICT}_{t}+{\sum_{j=1}^{K}\delta_{j}X}_{jt}+\varepsilon_{t}$$

Drawing upon the environmental Kuznets curve (EKC) framework, which suggests an inverted U-shaped relationship between environmental degradation and economic growth, a theoretical model can be proposed that explores the dynamic interplay between opportunity entrepreneurship and ICT diffusion as influential factors in promoting environmental sustainability:

(2) $$E_{t}={a_{0}+a_{1}Y}_{t}+{a_{2}Y}^{2}+{a_{3}OpE}_{t}+a_{4}{ICT}_{t}+{\sum_{j=1}^{K}\delta_{j}X}_{jt}+\varepsilon_{t}$$

From this standard EKC model and to check the validity of the two first hypotheses, the following specifications are estimated:

(3) $$E_{t}={a_{0}+a_{1}OpE}_{t}+{a_{2}OpE}^{2}+{a_{3}Y}_{t}+a_{4}{ICT}_{t}+{\sum_{j=1}^{K}\delta_{j}X}_{jt}+\varepsilon_{t}$$

(4) $$E_{t}={a_{0}+a_{1}ICT}_{t}+{a_{2}ICT}^{2}+{a_{3}Y}_{t}+a_{4}{OpE}_{t}+{\sum_{j=1}^{K}\delta_{j}X}_{jt}+\varepsilon_{t}$$

In Eq. (2), environmental degradation, represented by CO_2_ emissions and ecological footprint, is influenced by a series of factors, including per capita GDP (Y), squared GDP per capita (Y^2^), opportunity entrepreneurship (OpE), information and communication technology (ICT), and a set of control variables (X), including financial development, trade openness, and rule of law. Following the EKC model, which posits a nonlinear relationship between environmental degradation and economic growth, it is expected that the initial growth of the economy increases E (with ∂Y_t_/∂E_t_ ≥ 0) but eventually decreases it as the economy matures (∂Y_t_^2^/∂E_t_ < 0). This model is further nuanced by replacing Y^2^ with squared opportunity entrepreneurship (OpE^2^) in Eq. (3) and squared ICT (ICT^2^) in Eq. (4), each time examining the nonlinear effects of these variables on environmental degradation. These modifications suggest that while initial increases in OpE and ICT may worsen environmental conditions (∂OpE_t_/∂E_t_ ≥ 0, ∂ICT_t_/∂E_t_ ≥ 0), beyond a certain threshold, further development in these factors contributes to environmental improvement (∂OpE^2^/∂E_t_ < 0, ∂ICT^2^/∂E_t_ < 0).

The linear and non-linear effects of ICT diffusion and opportunity entrepreneurship on environmental quality imply established thresholds for realizing this. The values of these thresholds are calculated and presented in Table 6. Following Nchofoung et al. [37], this threshold can be calculated through Eq. (5):

(5) $$Threshold=\frac{Coefficient\;ICT\;\left(OpE\right)}{2\;X\;Coefficient\;ICT\left(OpE\right)\;squared}\;x\;100$$

To validate hypothesis 3 and consider the modulating effect of ICT on the nexus between opportunity entrepreneurship and CO_2_ emissions, ecological footprint, and EPI, it was rewritten Eq. (1) as follows:

(6) $$E_{t}=a_{0}^{+}{a_{1}OpE}_{t}+a_{2}{ICT}_{t}+\pi \left({ICT}_{t}\;x\;{OpE}_{t}\right)+{\sum_{j=1}^{K}\delta_{j}X}_{jt}+\varepsilon_{t}$$

where *ICT ∗ OpE* indicates the interaction between the proxies of ICT diffusion and the two types of indicators of environmental degradation. X denotes the control variables, including GDP per capita, financial development index, trade openness, and rule of law. As argued above, ICT diffusion is anticipated to moderate the negative impact of opportunity entrepreneurship on CO_2_ emissions and ecological footprint. π is the coefficient of the modulating variable. Differentiating Eq. (6) with respect to OpE yields Eq. (7) below:

(7) $$\frac{\partial E_{t}}{\partial {OpE}_{t}}=\alpha_{1}+\pi {ICT}_{t}$$

Where ∂ represents the partial derivative operator, a one-unit improvement in environmental quality due to variations in OpE depends on the signs and coefficients of α_1_ and π. The net effects can be calculated only when both the direct and indirect coefficients are significant and have opposite signs.

In the first step of the empirical analysis, a stationarity assessment of the variables is necessary, followed by demonstrating their cointegration to validate the long-term relationships prior to applying the dynamic ordinary least squares (DOLS) method to estimate long-run relationships. However, the advantages of the DOLS estimator are also discussed. For example, this estimator is capable of controlling for endogeneity through the addition of lagged versions of both dependent and independent variables in addition to feedback effects, improving the reliability of the estimates (Omri et al., 2019). Moreover, DOLS can also deal with short-term memory and heteroscedasticity in the data; thus, results are more precisely measured and forecasted. The approach enables the formation of lagged variables in its model, which is advantageous because the lagged variables are important not only for better specification but also for elucidating the underlying relationships among the variables. Through accounting for endogeneity, DOLS accounts for the interaction of independent and dependent variables and eliminates bias by enabling more accurate estimates. Furthermore, the ability to keep up with those estimations over time will contribute to the forecasting model's accuracy and credibility, making it possible for policymakers and researchers to rely on valid and trustworthy projections after that. The DOLS model can also deal with non-stationarity problems, including trends and structural breaks. This capacity of the model provides it with the advantage of being robust and adaptable. Hence, the model can detect different changes in the underlying dynamics and provide accurate forecasts that are dynamic.

The DOLS estimator is specified as follows, according to Stock and Watson (1993):

(8) $$Y_{t}=\beta +\vec{\beta }X+\sum_{j=-q}^{p}{\vec{d}}_{j}{\Delta X}_{t-j}+u_{t}$$

Where *Y*, *X*, *q*, and *p* represent the dependent variable, independent variables, lead length, and lag length, respectively.

### 3.2 Data

This study examines the influence of ICT diffusion and opportunity entrepreneurship on improving environmental quality. The period from 2009 to 2020 is covered in the analysis. Data for the study were collected from several sources, such as the 10.13039/100008086Global Entrepreneurship Monitor (GEM), the World Development Indicators (WDI), the 10.13039/100008086Global Footprint 10.13039/100031212Network (GFN), the International Monetary Fund (IMF), Our World in Data, and the Yale Center for Environmental Law and Policy (YCELP) online databases. Further details on the data description and sources are found in Table 1.

**Table 1:** Measurement of the variables and their sources.

Variable	Measurement	Source	Symbols	Expected signs *on*		
				CO	Ef	EP
CO_2_ emissions	Metric tons per capita	WDI	CO	*NA*	*NA*	*NA*
Ecological footprint	Global hectares per capita	GFN	Ef	*NA*	*NA*	*NA*
Environmental performance index (EPI)	EPI's score is normalized on a scale of 0–100.	YCELP	EPI	*NA*	*NA*	*NA*
Opportunity entrepreneurship	Percentage of individuals between the ages of 18 and 64 who initiate a new business to capitalize on a promising business opportunity	GEM	OpE	+	+	–
Mobile cellular subscription (Use)	Per 100 people	WDI	Access	+	+	–
Internet use (Access)	Percentage of population	WDI	Use	+	+	–
Mean years of schooling (Skills)	Average years of formal education for individuals aged 15–64 years	Our World in Data	Skills	+	+	–
GDP per capita	Constant 2015 US$	WDI	Y	+	+	–
Financial development	Financial development index	IMF	Fd	+/−	+/−	+/−
Trade openness	Percentage of GDP	WDI	Trade	+/−	+/−	+/−
Rule of law	Index ranging from approximately −2.5 to 2.5 (estimate)	WDI	RL	–	+	+

The dependent variable is environmental degradation, measured by CO_2_ emissions, ecological footprint, and environmental performance index. *CO*_*2*_ *emissions* represent the main indicator of environmental degradation since they show the amount of CO_2_ emitted into the atmosphere due to activities that include burning fossil fuels, industrial processes, and deforestation [38]. These emissions are the subject of the debate on climate change, evidencing the connection between human-caused activities and global warming. The measurement of CO_2_ emissions enables us to see how different economic and policy settings can either exacerbate or alleviate environmental damage [39]. Consistently with CO_2_ emissions, *ecological footprint* provides a more comprehensive measure of the degradation of the environment by evaluating the land and resource consumption of a population or an activity [40]. This indicator covers diverse environmental components, namely energy use, water use, waste generation, and land use, to determine whether the consumption patterns are ecologically sustainable in light of the Earth's regenerative capability [41]. In this way, the ecological footprint becomes a comprehensive indicator of how humans exceed the regeneration potential of resources and assimilation function for waste, thus highlighting the need for sustainable resource management and conservation [42]. Data on per capita CO_2_ emissions is sourced from the WDI online database, whereas data on per capita ecological footprint is obtained from the GFN online database. *Environmental performance index* classifies countries' performance on great-priority environmental problems in two broad policy fields: protecting human health from environmental stress and protecting ecosystems and natural resources (Esty et al., 2005; Emerson et al., 2022). Data from the 2022 EPI and additional details on data calculations and sources can be downloaded from https://epi.yale.edu/.

The independent variables of interest are opportunity entrepreneurship and ICT diffusion. *Opportunity entrepreneurship*, based on the GEM project, is a key element of a country's economic and innovative environment. Entrepreneurship in this form is related to persons who engage in new business ventures, exploiting opportunities instead of necessity or lack of employment [5,43]. The GEM project relies on the Total Early-Stage Entrepreneurial Activity (TEA) measure to characterize the proportion of 18-64-year-olds who own or manage a business, covering all the aspects of a country's entrepreneurial activity. Many reasons support the emphasis on opportunity-driven entrepreneurship, which explicitly reveals the active initiative-taking nature of entrepreneurs when they identify and pursue nascent market opportunities commonly linked to innovations, economic growth, and employment generation (Omri et al., 2024). Different from necessity-driven entrepreneurship, which arises out of the lack of alternatives, opportunity entrepreneurship is characterized as a deliberate choice to pursue business ideas perceived as being lucrative or promising [44]. Opportunity entrepreneurs are typically a major source of competitive advantage for economies as they bring new products, services, and technologies to the market, encouraging dynamism and resilience within the economic system [43]. Data on opportunity entrepreneurship were collected from the GEM online database. On the other hand, *ICT diffusion* is assessed through three proxies—access, usage, and skills—measured by mobile cellular subscriptions per 100 persons, internet usage percentage, and average years of schooling, as identified by Avom et al. [45] and Ofori and Asongo [46]. In addition, research has shown that ICTs contribute significantly to enhancing entrepreneurial activities [47], stimulating growth of economy [48], and promoting environmental protection measures [49].

Consistent with the existing sustainability literature, the analysis includes several control variables: per capita GDP, trade openness, financial development, and rule of law. Economic growth, reflected in GDP per capita, exhibits a complex relationship with environmental sustainability. Although higher GDP per capita usually implies more resources to invest in sustainable technologies and environmental protection, this also means higher consumption and more significant environmental degradation [50,51]. Financial development is expected to contribute to environmental sustainability through investments in green technologies and sustainable practices, as supported by Omri et al. (2021) and Ben Youssef et al. [52]. Moreover, as Kahia et al. [53,54] studied, trade openness is argued to advance environmental sustainability through disseminating environmentally friendly technologies and practices. Additionally, the rule of law is fundamental in maintaining environmental sustainability through upholding environmental regulations and governance standards, consistent with the findings of Ben Youssef et al. (2018) and Nchofoung and Asongu [55].

## 4 Results and discussion

The summary statistics and the results of pair-wise correlations are reported in Table 2, Table 3, respectively. Notably, the CO_2_ emissions range from 16.563 to 19.375 metric tons per capita across the sample period, with an average of 17.348. Ecological footprint ranges from 5.029 to 6.853 ha per capita, averaging 5.860. The score of EPI spans from 37.7 to 68.36, with an average score of 60.054, where a higher score means better performance. Regarding ICT components, over 145 mobile phones are used by every 100 people, and over 70 % of the population has internet access. Regarding ICT skills, the average number of years of schooling stands at approximately 9.5 years, ranging from approximately 8.5 to 10 years. Regarding the entrepreneurship variable, the percentage of individuals involved in opportunity-driven entrepreneurship ranges from 69.68 to 95.38 %, with an average of 80.288 %. Table 3 displays the correlations between variables, illustrating the linear relationship between the two variables. The study examines the association between independent variables concerning entrepreneurship and ICT diffusion and their linearity with the dependent variables, CO_2_ emissions, ecological footprint, and EPI. The correlation results align with expectations, indicating a negative association between opportunity entrepreneurship, ICT's three components, and environmental quality indicators. Opportunity entrepreneurship, ICT use, access, and skills are positively correlated with CO_2_ emissions and ecological footprint and negatively correlated with PPE. This means that higher entrepreneurial activity and ICT diffusion levels can contribute to environmental degradation, as evidenced by increased CO_2_ emissions and the ecological footprint, while leading to lower environmental performance. Furthermore, opportunity entrepreneurship is highly and positively correlated with ICT use, access, and skills, meaning that the integration of ICT tools and resources into the entrepreneurial ecosystem has enabled more individuals to discover and pursue promising business opportunities, leading to higher rates of opportunity-motivated entrepreneurship than those driven by necessity. Thus, it is clear that ICT helps to engage more people in entrepreneurial activity as it offers means of communication, market research, networking facilities, and resource management that empower individuals to see the possibilities and contribute to the economy through innovation and growth. At the same time, it should be noted that these correlations can indicate only an initial direction of the impact of the independent variables on the dependent variable. In-depth analysis is required to attain deeper insight, as the correlation coefficients only show the extent of the linear nexus between each set of variables. This assumption is explored further within the research by exploiting the multivariate regression through the cointegration approach coupled with long-term estimates that offer detailed interplay relations between variables.

**Table 2:** Descriptive statistics.

Variable	Mean	SD	Min	Max
CO_2_ emissions	17.348	0.986	16.563	19.375
Ecological footprint	5.860	0.506	5.029	6.853
Environmental performance	60.054	11.445	37.7	68.36
Opportunity entrepreneurship	80.288	8.692	69.48	95.38
Mobile cellular subscription (Use)	147.586	24.843	115.271	179.099
Internet use (Access)	71.637	22.731	38	100
Mean years of schooling (Skills)	9.564	0.553	8.592	10.225
GDP per capita	75024.847	4172.425	66204.1429	79530.4423
Financial development	0.459	0.0357	0.397	0.519
Trade openness	70.526	12.586	49.713	84.861
Rule of law	0.165	0.008	0.154	0.181

Note: SD, Min, and Max indicate standard deviations, minimum and maximum. (Source: authors elaboration).

**Table 3:** Correlations matrix.

Variables	1.	2.	3.	4.	5.	6.	7.	8.	9.	10.	11.
1. CO_2_ emissions	1										
2. Ecological footprint	0.715	1									
3. Environmental performance	−0.495	−0.034	1								
4. Opportunity entrepreneurship	0.707	0.651	−0.084	1							
5. Mobile cellular subscription (Use)	0.485	0.505	−0.679	0.776	1						
6. Internet use (Access)	0.337	0.817	−0.582	0.667	0.699	1					
7. Mean years of schooling (Skills)	0.425	0.866	−0.520	0.738	0.651	0.694	1				
8. GDP per capita	0.562	0.636	−0.325	0.700	0.675	0.558	0.773	1			
9. Financial development	0.441	0.293	0.482	0.172	0.113	−0.041	−0.021	0.199	1		
10. Trade openness	0.548	0.630	0.337	0.790	0.585	0.661	0.775	0.599	−0.195	1	
11. Rule of law	−0.330	−0.671	0.345	0.782	0.281	0.552	0.600	0.766	0.724	0.760	1

Before proceeding with the empirical model estimation, Two-unit root tests were conducted, namely the Augmented Dickey-Fuller (ADF) and Philippe and Perron (PP) tests, to evaluate the stationarity of the variables under consideration. The results in Table 4 show that most variables are non-stationary at their original levels but stabilize and achieve stationarity after first-order differencing. This suggests that the variables are first-order integrated. Next, Johansen's [56] cointegration test was applied to examine the long-run relationships among the used variables. The findings, reported in Table 5, reveal that the trace statistics exceed the critical values at rank = 0, demonstrating that all models did not reject the hypothesis of cointegration. Consequently, DOLS long-term estimations were assessed. The results are presented in Table 6, Table 7.

**Table 4:** Results of the unit root tests.

Variables	ADF		PP	
	Level	Δ	Level	Δ
CO_2_ emissions	−2.003	−2.139∗∗	−1.929	−2.100∗∗
Ecological footprint	−1.392	−4.476∗∗∗	−1.391	−4.472∗∗∗
Environmental performance index	−5.609∗∗∗	−5.576∗∗∗	0.287	−3.269∗∗
Opportunity entrepreneurship	−2.225	−6.017∗∗∗	−0.815	−5.640∗∗∗
Mobile cellular subscription (Use)	−1.061	−4.841∗	−6.375∗∗∗	−3.457∗∗∗
Internet use (Access)	−4.261∗∗∗	−4572∗∗∗	−7.208∗∗∗	−3.759∗∗∗
Mean years of schooling (Skills)	−2.567∗	−2.574∗∗	−7185∗∗∗	−2.574∗∗
GDP per capita	−1.248	−3.906∗	−1.276	−3.865∗∗∗
Financial development	−1.864	−4.137∗∗	−1.864	−4.127∗∗
Trade openness	−0.687	−4.510∗	−0.873	−3.283∗∗
Rule of law	−3.719∗∗	−4.445∗∗∗	−1.658	−4.422∗∗∗

Note: ∗, ∗∗, and ∗∗∗ show the significance at 1 %, 5 %, and 10 % levels, respectively. Δ indicates first differences. ADF and PP indicate Augmented Dickey-Fuller and Philippe and Perron. (Source: authors elaboration).

**Table 5:** Results of Johansen's cointegration test.

	Rank = 0∗	OpE		Use		Access		Skills	
		Trace test	Critical value (5 %)	Trace test	Critical value (5 %)	Trace test	Critical value (5 %)	Trace test	Critical value (5 %)
CO	0	141.037	95.753	160.540	95.753	143.155	95.753	137.570	95.753
Ef	0	153.403	95.753	163.218	95.753	154.762	95.753	128.399	95.753
EPI	0	172.471	95.753	168.200	95.753	159.650	95.753	131.770	95.753

Note: OpE indicates opportunity entrepreneurship. CO, Ef, and EPI are carbon emissions, ecological footprint, and environmental performance index, respectively. Use, access, and skills indicate internet use, access, and skills. (Source: authors elaboration).

**Table 6:** Non-linear effects of entrepreneurship and ICT on CO_2_ emissions, Ecological Footprint, and Environmental Performance Index.

Variables	CO_2_ emissions				Ecological footprint				Environmental Performance Index (EPI)			
	OpE	ICT diffusion			OpE	ICT diffusion			OpE	ICT diffusion		
		Model 1	Model 2	Model 3		Model 1	Model 2	Model 3		Model 1	Model 2	Model 3
OpE	0.017∗∗∗	–	–	–	0.016∗∗∗	–	–	–	−0.021∗∗	–	–	–
	(0.000)				(0.000)				(0.012)			
OpE^2^	−0.139∗∗∗	–	–	–	−0.135∗∗∗	–	–	–	0.104	–	–	–
	(0.000)				(0.000)				(0.124)			
ICT use (Use)	–	0.015∗∗	–	–	–	0.018∗∗	–	–	–	−0.013∗∗∗	–	–
		(0.019)				(0.028)				(0.000)		
ICT access (Access)	–	–	0.011∗∗∗	–	–	–	0.012∗	–	–	–	−0.015∗∗∗	–
			(0.000)				(0.062)				(0.000)	
ICT skills (Skills)	–	–	–	0.008∗	–	–	–	0.003∗	–	–	–	−0.013∗∗∗
				(0.054)				(0.050)				(0.000)
Use^2^	–	−0.093∗∗	–	–	–	−0.102∗∗∗	–	–	–	0.083	–	–
		(0.036)				(0.000)				(0.139)		
Access^2^	–	–	−0.109∗∗∗	–	–	–	−0.114∗∗	–	–	–	0.103	–
			(0.002)				(0.010)				(0.111)	
Skills^2^	–	–	–	−0.298∗∗∗	–	–	–	−0.182∗∗∗	–	–	–	0.053
				(0.000)				(0.000)				(0.236)
Thresholds	87.987	173.430	92.58	9.008	87.791	173.010	92.33	9.056	n.a.	n.a.	n.a.	n.a.
GDP per capita	0.329∗∗∗	0.346∗∗∗	0.467∗∗∗	0.294∗∗∗	0.271∗∗∗	0.318∗∗∗	0.342∗∗∗	0.266∗∗∗	−0.216∗∗∗	−0.294∗∗∗	−0.311∗∗∗	−0.305∗∗∗
	(0.000)	(0.000)	(0.000)	(0.000)	(0.000)	(0.000)	(0.000)	(0.000)	(0.000)	(0.000)	(0.000)	(0.000)
Financial development	0.094∗∗	0.110∗∗∗	0.089	0.107∗∗	0.122∗∗∗	−0.079	0.083	0.134∗∗∗	−0.111∗∗	−0.138∗∗∗	−0.101	−0.125∗∗
	(0.011)	(0.000)	(0.108)	(0.032)	(0.000)	(0.126)	(0.110)	(0.000)	(0.019)	(0.000)	(0.108)	(0.020)
Trade openness	0.191∗∗∗	0.219∗∗∗	0.188∗∗∗	0.241∗∗∗	0.154∗∗	0.293∗∗∗	0.311∗∗∗	0.298∗∗∗	0.166∗∗∗	−0.193∗∗∗	−0.158∗∗∗	−0.171∗∗∗
	(0.000)	(0.000)	(0.000)	(0.000)	(0.029)	(0.000)	(0.000)	(0.000)	(0.000)	(0.000)	(0.000)	(0.000)
Rule of law	−0.093∗∗∗	−0.129∗∗∗	−0.099∗∗∗	−0.102∗∗	−0.114∗	−0.122∗∗∗	−0.114∗∗∗	−0.121∗∗∗	0.096∗∗	0.111∗∗	0.120∗∗	0.119∗∗∗
	(0.001)	(0.000)	(0.000)	(0.023)	(0.062)	(0.000)	(0.000)	(0.000)	(0.030)	(0.016)	(0.010)	(0.009)
Constant	0.739∗∗∗	1.839∗∗∗	1.239∗∗∗	0.830∗∗∗	0.508∗∗∗	0.930∗∗∗	1.104∗∗∗	0.922∗∗∗	0.711∗∗∗	0.839∗∗∗	0.617∗∗∗	0.799∗∗∗
	(0.000)	(0.000)	(0.000)	(0.000)	(0.000)	(0.000)	(0.000)	(0.000)	(0.000)	(0.000)	(0.000)	(0.000)

Note: The dependent variables are CO_2_ emissions, ecological footprint, and EPI. Coefficients are estimated using the DOLS estimator. P-values are given in parentheses. ∗, ∗∗, and ∗∗∗ indicate the significance at 10 %, 5 %, and 1 % levels, respectively. Models 1 to 3 indicate ICT use, ICT access, and ICT skills, respectively. OpE indicates opportunity entrepreneurship. (Source: authors elaboration).

**Table 7:** Complementarity effects of entrepreneurship and ICT on both CO_2_ emissions and ecological footprint.

Variables	CO_2_ emissions			Ecological footprint			Environmental Performance Index (EPI)		
	ICT diffusion			ICT diffusion			ICT diffusion		
	Model 1	Model 2	Model 3	Model 1	Model 2	Model 3	Model 1	Model 2	Model 3
OpE	0.191∗∗∗	0.179∗∗∗	0.204∗∗∗	0.169∗∗∗	0.150∗∗	0.193∗∗∗	−0.097∗∗	−0.118∗∗	−0.144∗∗∗
	(0.000)	(0.000)	(0.000)	(0.000)	(0.010)	(0.000)	(0.031)	(0.022)	(0.000)
ICT use (Use)	0.094∗	–	–	0.117∗∗	–	–	−0.092∗∗	–	–
	(0.052)			(0.028)			(0.040)		
ICT access (Access)	–	0.133∗∗∗	–	–	0.164∗	–	–	−0.128∗	–
		(0.000)			(0.000)			(0.062)	
ICT skills (Skills)	–	–	0.121∗∗	–	–	0.093∗∗	–	–	−0.156∗∗∗
			(0.014)			(0.018)			(0.000)
Use x OpE	−0.019∗∗∗	–	–	−0.009∗∗∗	–	–	0.014	–	–
	(0.000)			(0.000)			(0.000)		
Access x OpE	–	−0.024∗∗∗	–	–	−0.023∗∗∗	–	–	0.013∗∗∗	–
		(0.000)			(0.000)			(0.000)	
Skills x OpE	–	–	−0.048∗∗∗	–	–	−0.056∗∗∗	–	–	0.024∗∗∗
			(0.000)			(0.000)			(0.000)
**Net effects**	−2.613	−1.540	−0.255	−1.159	−1.497	−0.342	n.a.	0.813	0.085
GDP per capita	0.280∗∗∗	0.222∗∗∗	0.195∗∗∗	0.279∗∗∗	0.207∗∗∗	0.205∗∗∗	−0.244∗∗∗	−0.252∗∗∗	−0.207∗∗∗
	(0.000)	(0.000)	(0.000)	(0.000)	(0.000)	(0.000)	(0.000)	(0.000)	(0.000)
Financial development	0.171∗∗∗	0.156∗∗	0.143∗∗	0.102	0.151∗∗∗	0.158∗∗∗	−0.111	−0.120∗	−0.132∗
	(0.033)	(0.028)	(0.020)	(0.117)	(0.002)	(0.001)	(0.110)	(0.059)	(0.062)
Trade openness	0.221∗∗∗	0.192∗∗∗	0.183∗∗∗	0.199∗∗∗	0.179∗∗∗	0.169∗∗	−0.159∗∗∗	−0.191∗∗∗	−0.178∗∗∗
	(0.000)	(0.000)	(0.000)	(0.000)	(0.000)	(0.026)	(0.000)	(0.000)	(0.000)
Rule of law	−0.127∗∗∗	−0.098∗∗∗	−0.111∗∗∗	0.089∗∗	−0.129∗∗∗	−0.118∗∗	0.087∗∗∗	0.134∗∗∗	0.159∗∗∗
	(0.000)	(0.000)	(0.000)	(0.012)	(0.000)	(0.022)	(0.000)	(0.000)	(0.000)
Constant	0.930∗∗∗	1.078∗∗∗	0.843∗∗∗	0.917∗∗∗	0.984∗∗∗	0.893∗∗∗	0.638∗∗∗	0.568∗∗∗	0.596∗∗∗
	(0.000)	(0.000)	(0.000)	(0.000)	(0.000)	(0.000)	(0.000)	(0.000)	(0.000)

Note: The dependent variables are CO_2_ emissions, ecological footprint, and EPI. Coefficients are estimated using the DOLS estimator. P-values are given in parentheses. ∗, ∗∗, and ∗∗∗ indicate the significance at 10 %, 5 %, and 1 % levels, respectively. The mean values of ICT use, ICT access, and ICT skills are 147.586, 71.637, and 9.564, respectively. n.a. indicates not applicable because at least one of the coefficients used in the calculation of the net effect is not significant. Models 1 to 3 indicate ICT use, ICT access, and ICT skills, respectively. OpE indicates opportunity entrepreneurship. (Source: authors elaboration).

Table 6 shows the results of the linear and non-linear impacts of both opportunity entrepreneurship and the proxies of ICT diffusion on the indicators of environmental quality. Opportunity entrepreneurship is found to have significant effects on increasing CO_2_ emissions (0.171) and ecological footprint (0.189), whereas its effects on environmental performance are negative (−0.134). Specifically, for every unit upsurge in opportunity entrepreneurship, carbon emissions are expected to increase by 0.171 metric tons per capita, while ecological footprint is expected to increase by 0.189 ha per capita. In contrast, the effects of opportunity entrepreneurship on environmental performance, as measured by the EPI, are negative, suggesting that higher levels of opportunity entrepreneurship are associated with lower environmental performance. The negative effects of entrepreneurial activity, particularly opportunity-driven entrepreneurship, on the quality of the environment confirm the results of Omri and Afi [5], who examined the associations between education and four distinct types of entrepreneurship—informal, formal, necessity, and opportunity entrepreneurship—and the sectoral dimension of environmental sustainability across 32 developing nations. They found that entrepreneurial activities contribute to environmental degradation, with informal entrepreneurship and necessity entrepreneurship exerting the most significant impact on air pollution. The same result was found by Dhahri et al. [4], who used data from 20 developing countries to reinvestigate the effect of necessity and opportunity entrepreneurship on sustainable development (in its three dimensions). The authors demonstrated that both entrepreneurship types negatively influence the environmental dimension, and opportunity entrepreneurship has a smaller impact on environmental degradation than necessity entrepreneurship. Their findings echo the worrying trend observed by Omri and Afi [5], highlighting the negative influence of entrepreneurial activities on environmental well-being. Notably, even though opportunity entrepreneurship demonstrated a comparatively lesser degree of environmental impact than its necessity-driven counterpart, the overall conclusion remains clear: entrepreneurial efforts, regardless of their motivation, tend to exacerbate environmental degradation, posing significant challenges to achieving sustainable development goals. These studies collectively highlight the imperative to reassess entrepreneurship's role in environmental sustainability and explore strategies to mitigate its negative effects while promoting innovation and economic growth. However, the coefficients of the OpE^2^ (squared opportunity entrepreneurship) are statistically negative for CO_2_ emissions (−0.119) and ecological footprint (−0.132) and positive for the EPI (0.122). The observed quadratic relationship between environmental degradation and opportunity entrepreneurship, as evidenced by the positive and negative coefficients of the linear and non-linear terms, aligns with previous research findings. For instance, Omri [15] investigated this relationship across 69 countries categorized into income-based panels, revealing that while opportunity entrepreneurship initially degrades environmental quality, it eventually improves it after reaching a certain threshold, indicating an inverted U-shaped relationship. This non-linear pattern is consistent with the EKC hypothesis, which suggests that environmental degradation first increases with economic growth but eventually decreases once a certain level of development is reached. The negative coefficients for CO_2_ emissions and ecological footprint suggest that higher levels of opportunity entrepreneurship are associated with reduced environmental impact, supporting the notion that entrepreneurship can drive sustainable development (Carayannis et al., 2015). However, the positive coefficient for the EPI implies that despite initial degradation, opportunity entrepreneurship ultimately leads to enhanced environmental performance, corroborating the argument that entrepreneurship fosters innovation and efficiency in resource use, ultimately benefiting the environment (Fernando & Lawrence, 2014). Despite the non-linearity, the dominance of the coefficient of OpE over that of OpE^2^ suggests that while immediate impacts on environmental sustainability may be limited, sustained increases in opportunity entrepreneurship could lead to significant long-term improvements in environmental quality, supporting the need for continued support and encouragement of entrepreneurial activity for sustainable development (Sarkis et al., 2018).

Moreover, Table 6 also shows that across various ICT proxies and environmental measures, ICT exhibits negative effects on environmental quality, ranging from 0.008 % (skills) to 0.015 % (use) on CO_2_ emissions, from 0.003 % (skills) to 0.018 % (use) on ecological footprint, and from −0.013 % (use) to −0.015 % (access) on EPI. Specifically, for every unit increase in ICT, CO_2_ emissions are expected to increase by 0.008–0.015 metric tons per capita, and ecological footprint is expected to increase by 0.003–0.018 ha per capita. On the other hand, the impact of ICT on environmental performance, as measured by the EPI, is negative, suggesting that higher levels of ICT are associated with lower environmental performance. ICT diffusion can negatively impact environmental quality through different means. First, ICT products' production, usage, and disposal cause environmental degradation. The process of manufacturing ICT devices consumes much energy and materials, which results in the emission of greenhouse gases and pollutants. Moreover, the harmful disposal of toxic waste from old devices may cause soil and water contamination, threatening the ecological system [57]. Along with this, the infrastructure operation of ICT, such as data centers and servers, contributes to carbon emissions and higher energy consumption of electricity [8,58]. Additionally, due to the proliferation of ICT networks and services, the need for electricity for network devices and cooling systems increases [59]. Furthermore, the introduction of ICT by most households can also indirectly affect the environment through changing consumption patterns and how people behave. This is demonstrated through, for example, online shopping and digital entertainment, which promote higher consumption levels and expand the need for resource extraction, production, and waste generation [60]. The negative impacts of ICT on environmental quality partially contradict the findings of Khan et al. (2020), who explored the nexus between ICT and CO_2_ emissions across 91 developed and developing countries, and they found that, overall, ICT has a mitigating effect on carbon emissions across all countries examined. However, the comparative analysis between developed and developing nations unveils a contrasting pattern, indicating that while ICT promotes environmental sustainability in developed countries, its impact is less favorable in developing countries. Similarly, Appiah-Otoo et al. (2022) also examined, using panel data from 110 countries, the impact of ICT on environmental degradation, considering differences in ICT quality among countries. The authors found that ICT improved environmental sustainability in economies with higher ICT quality. However, in economies with low and moderate ICT quality, the authors observed a detrimental impact of ICT on the environment, leading to increased environmental degradation. The authors recommend policies to promote ICT usage through affordable pricing in moderate and low ICT-quality countries to improve environmental quality. More efforts to bridge the digital divide and improve access to affordable ICT services in developing countries should be complemented with initiatives to promote environmental sustainability. This includes integrating environmental education and awareness into ICT training programs and leveraging ICT for monitoring and managing environmental resources more effectively. In contrast to the negative linear effects of the ICT proxies on the indicators of environmental quality, the coefficients of the ICT^2^ (squared ICT) are statistically negative on per capita CO_2_ emissions and ecological footprint, ranging from −0.093 % (use) to −0.298 % (skills) for CO_2_ emissions and from −0.102 % (use) to −0.182 % (skills) for ecological footprint. This indicates a quadratic relationship, where the impact of ICT on environmental indicators initially decreases but then remains flat or even reverses at higher levels of ICT. The negative coefficients of the squared ICT term suggest that an additional increase in ICT may lead to increased environmental impact beyond a certain point. This quadratic relationship between ICT and environmental indicators is consistent with previous research findings. The inverted U-shaped relationship between ICT proxies and CO_2_ emissions and ecological footprint aligns with previous research findings. For instance, Higón et al. [26] investigated the non-linear association between ICT and CO_2_ emissions worldwide, examining this connection across nations with varying levels of development. Their findings confirmed the inverted U-shaped relationship between CO_2_ emissions and ICT. In addition, the authors observed that the turning point (threshold) for ICT in developing countries surpassed the mean value, while in developed countries, it was the opposite. This suggests that numerous developed nations have reached a stage of ICT development where further advancements lead to a decline in CO_2_ emissions. Similarly, a study by Asongu et al. (2018) examined the nexus between CO_2_ emissions and ICT usage in 44 sub-Saharan African nations. The results showed that higher levels of ICT usage initially led to greater CO_2_ emissions per capita. However, they also determined policy threshold levels for ICT adoption, below which the net effects on emissions would remain positive. Specifically, they computed minimum standards of ICT diffusion that Saudi Arabia would need to surpass for digital technologies to start reducing CO_2_ collectively rather than increasing it. This threshold concept quantifies the point at which the quadratic relationship between ICT and emissions theoretically shifts from an upward-sloping to a downward-sloping trajectory. By identifying these threshold levels, policymakers can guide ICT adoption strategies to achieve optimal environmental outcomes. Therefore, investments in green ICT initiatives and sustainable practices can help mitigate the environmental footprint of ICT use, thereby contributing to overall sustainability goals. The results show that the thresholds obtained for the various ICT indicators are within the ICT ranges reported in the summary statistics. These thresholds thus have policy meaning, confirming a non-linear effect of ICT diffusion on environmental quality. Enhancing ICT increases environmental improvement up to this threshold (173 mobile cellular subscriptions per 100 people for ICT use, 92 percent of the population for internet access, and 9 years of schooling for ICT skills).

While the above discussion provides insights into the individual effects of opportunity entrepreneurship and ICT diffusion on environmental quality, it does not delve into the intricate dynamics between these variables. A critical gap exists in understanding how opportunity entrepreneurship and ICT diffusion interact synergistically or antagonistically to shape environmental outcomes. Specifically, the mechanisms underlying the joint effects of these factors on environmental sustainability remain largely unexplored. Therefore, the focus was on another gap in the existing studies, i.e., how variations in ICT diffusion levels may either exacerbate or alleviate the environmental repercussions of entrepreneurial activities. By addressing this gap, a deeper knowledge of the complex interactions between opportunity entrepreneurship and ICT dissemination can be obtained which makes governmental interventions and environmental stewardship initiatives more effective. Building upon the studies conducted by Afi et al. [43] and Kahia et al. [61], the overall effects of the interactions between the opportunity entrepreneurship and the proxies of ICT on both per capita CO_2_ emissions, per capita ecological footprint and EPI are evaluated by calculating net effects. In the case of each estimated model, a positive (negative) net effect confirms the validation of the tested hypothesis for CO_2_ emissions and ecological footprint (EPI), while a negative (positive) net effect signifies the rejection of the tested hypothesis for ecological footprint and CO_2_ emissions (EPI). Table 7 shows the negative net effects of the interplay between the three proxies of ICT and opportunity entrepreneurship on CO_2_ emissions and ecological footprint. In contrast, positive net effects on EPI are shown in the interaction between EPI and both ICT access and ICT skills. For example, in the third column (model 3) of Table 7, the net effect on per capita CO_2_ emissions from the interaction between ICT skills and opportunity entrepreneurship is −0.255[(−0.048 × 9.564) + (0.204)]. Similarly, in the ninth column (model 3) of Table 7, the net effect on the environmental performance index from the interaction between ICT skills and opportunity entrepreneurship is 0.0058 [(0.024 × 9.564) + (−0.144)]. In these formulas, 9.564 is the mean value of ICT skills, −0.048 and 0.024 are the marginal effects on, respectively, per capita CO_2_ emissions and EPI from increasing ICT skills, and 0.204 and −0.144 are the unconditional effects of opportunity entrepreneurship on per capita CO_2_ emissions and EPI, respectively. These positive net effects on environmental quality, as hypothesized, highlight the importance of the role of ICT diffusion in moderating the effect of opportunity entrepreneurship on environmental outcomes. This finding implies that in regions characterized by wide diffusion of ICT, the contributions of opportunity-driven entrepreneurs tend to translate into improved environmental conditions for the entire population. In essence, higher levels of ICT diffusion act as a facilitative mechanism, ensuring that the environmental consequences of entrepreneurial efforts tend toward positive outcomes. This dynamic suggests that the wide availability and adoption of ICT enables opportunity-driven entrepreneurship to more effectively align with environmental sustainability goals. Therefore, in environments where ICT penetration is high, entrepreneurial activities are more likely to contribute to the improvement of the environment rather than its degradation. This highlights the crucial interaction between ICT diffusion and entrepreneurial behavior in shaping environmental sustainability outcomes, highlighting the need for holistic approaches that take into account the complementarity of these factors to foster environmentally friendly economic development. Therefore, the diffusion of ICT holds great promise for opportunity-driven entrepreneurs, particularly for advancing environmental sustainability. First, it facilitates the rapid identification and exploitation of environmentally friendly business opportunities, enabling entrepreneurs to align their businesses with environmental goals (Zhang and Linag, 2012). Second, by fostering innovation, ICT contributes to the development of sustainable solutions and practices, thus promoting environmentally friendly entrepreneurship [11,25]. Additionally, ICT ecosystems provide fertile ground for the cultivation of green ideas and initiatives, thereby nurturing a culture of environmental awareness among entrepreneurs [62]. Finally, through improved connectivity and access to business information, ICT enables entrepreneurs to make informed decisions that prioritize environmental sustainability, thereby promoting the adoption of environmentally friendly practices and technologies [63].

Regarding the control variables, GDP per capita, financial development, and trade openness consistently exhibit negative associations with environmental quality across the estimated models, whereas the presence of a robust legal framework, as indicated by rules of law, tends to enhance environmental quality. The negative impacts of GDP per capita, financial development, and trade openness on the environmental quality indicate that advanced levels of economic activity and globalization might cause deterioration of the environment. These results are consistent with previous studies that find greater volumes of economic development and international trade lead to increased exploitation of resources, pollution, and habitat destruction ([50,52]; Omri et al., 2023). Furthermore, the positive impact of the rule of laws on environmental quality shows how important institutional quality and governance structures are in attaining environmental sustainability. Strong legal institutions are paramount for enforcing environmental standards, ensuring compliance with environmental standards, and holding polluters accountable ([64]; Omri and Boubaker, 2024). Countries with strong legal systems are better positioned to manage natural resources skillfully and regulate the operation of externalities that affect the environment [65].

Finally, to ensure the robustness of the results, we employed the Fully Modified Ordinary Least Square (FMOLS) estimator, which is recommended for addressing issues of endogeneity and serial correlation in panel data analysis. The results obtained from the FMOLS method, as shown in Table 8, Table 9, revealed several important findings. First, regarding the linear and non-linear effects of ICT and opportunity entrepreneurship on the three indicators of environmental quality, we find that ICT exhibits negative effects on environmental quality, ranging from 0.002 % (skills) to 0.011 % (use) on CO_2_ emissions, from 0.004 % (skills) to 0.023 % (use) on ecological footprint, and from −0.010 % (skills) to −0.021 % (access) on EPI (see Table 8). Specifically, for every unit increase in ICT, CO_2_ emissions are expected to increase by 0.002–0.011 metric tons per capita, and ecological footprint is expected to increase by 0.004–0.023 ha per capita. On the other hand, the impact of ICT on environmental performance, as measured by the EPI, is negative, suggesting that higher levels of ICT are associated with lower environmental performance. Moreover, a 1 % increase in opportunity entrepreneurship increases CO_2_ emissions and ecological footprint by 0.017 % and 0.016 %, respectively; however, its impact on the environmental performance index is negative. In contrast to the negative linear effects of the ICT proxies and opportunity entrepreneurship on the indicators of environmental quality, the coefficients of the squared values of ICT and opportunity entrepreneurship are statistically negative on per capita CO_2_ emissions and ecological footprint, indicating a quadratic relationship, where the impact of ICT and opportunity entrepreneurship on environmental indicators initially decreases but then remains flat or even reverses at higher levels of ICT and opportunity entrepreneurship. The negative coefficients of the squared values suggest that an additional increase in ICT and opportunity entrepreneurship may lead to increased environmental impact beyond a certain point (173 mobile cellular subscriptions per 100 people for ICT use, 92 percent of the population for internet access, and 9 years of schooling for ICT skills). Second, in addition to the non-linear effects of ICT and opportunity entrepreneurship on environmental quality, we also show that the complementarity between ICT proxies and opportunity entrepreneurship yield mixed effects, with negative net effects on CO_2_ emissions and ecological footprint countered by positive net effects on the environmental performance index. These findings highlight the dual role of ICT diffusion as a contributor to environmental challenges and a potential solution, depending on the level of its diffusion and interaction with entrepreneurial activities. Overall, the results obtained through the FMOLS method confirm the findings obtained using the DOLS method, highlighting the non-linear effects of both ICT diffusion and opportunity entrepreneurship on environmental quality. It also confirms the complementarity relationship between ICT diffusion and opportunity entrepreneurship in improving environmental quality.

**Table 8:** Robustness check: Non-linear effects of entrepreneurship and ICT on CO_2_ emissions, Ecological Footprint, and EPI using FMOLS estimator.

Variables	CO_2_ emissions				Ecological footprint				Environmental Performance Index (EPI)			
	OpE	ICT diffusion			OpE	ICT diffusion			OpE	ICT diffusion		
		Model 1	Model 2	Model 3		Model 1	Model 2	Model 3		Model 1	Model 2	Model 3
OpE	0.007∗∗∗	–	–	–	0.010∗∗∗	–	–	–	−0.003∗∗∗	–	–	–
	(0.000)				(0.000)				(0.001)			
OpE^2^	−0.089∗∗∗	–	–	–	−0.107∗∗∗	–	–	–	0.038	–	–	–
	(0.000)				(0.000)				(0.113)			
ICT use (Use)	–	0.011∗∗∗	–	–	–	0.023∗∗∗	–	–	–	−0.019∗∗∗	–	–
		(0.000)				(0.000)				(0.000)		
ICT access (Access)	–	–	0.009∗∗∗	–	–	–	0.016∗∗∗	–	–	–	−0.021∗∗∗	–
			(0.000)				(0.000)				(0.000)	
ICT skills (Skills)	–	–	–	0.002∗∗∗	–	–	–	0.004∗∗∗	–	–	–	−0.010∗∗∗
				(0.000)				(0.000)				(0.008)
Use^2^	–	−0.083∗∗∗	–	–	–	−0.115∗∗∗	–	–	–	0.006	–	–
		(0.000)				(0.000)				(0.256)		
Access^2^	–	–	−0.099∗∗∗	–	–	–	−0.132∗∗∗	–	–	–	0.010	–
			(0.000)				(0.000)				(0.134)	
Skills^2^	–	–	–	−0.015∗∗∗	–	–	–	−0.021∗∗∗	–	–	–	0.013
				(0.000)				(0.000)				(0.122)
**Thresholds**	**88.370**	**174.190**	**91.82**	**8.888**	**87.341**	**173.913**	**91.83**	**9.070**	**n.a.**	**n.a.**	**n.a.**	**n.a.**
GDP per capita	0.298∗∗∗	0.364∗∗∗	0.390∗∗∗	0.369∗∗∗	0.291∗∗∗	0.303∗∗∗	0.292∗∗∗	0.283∗∗∗	−0.342∗∗∗	−0.290∗∗∗	−0.277∗∗∗	−0.283∗∗∗
	(0.000)	(0.000)	(0.000)	(0.000)	(0.000)	(0.000)	(0.000)	(0.000)	(0.000)	(0.000)	(0.000)	(0.000)
Financial development	0.127∗∗∗	0.119∗∗∗	0.123∗∗	0.097∗	0.103	0.139∗∗∗	0.111∗∗	0.140∗∗∗	−0.130∗∗∗	−0.127∗∗∗	−0.138∗∗	−0.155∗∗∗
	(0.000)	(0.000)	(0.017)	(0.050)	(0.120)	(0.000)	(0.019)	(0.000)	(0.000)	(0.000)	(0.017)	(0.000)
Trade openness	0.231∗∗∗	0.290∗∗∗	0.283∗∗∗	0.267∗∗∗	0.199∗∗∗	0.214∗∗∗	0.229∗∗∗	0.238∗∗∗	0.196∗∗∗	−0.225∗∗∗	−0.240∗∗∗	−0.218∗∗∗
	(0.000)	(0.000)	(0.000)	(0.000)	(0.000)	(0.000)	(0.000)	(0.000)	(0.000)	(0.000)	(0.000)	(0.000)
Rule of law	−0.079∗∗∗	−0.088∗∗∗	−0.103∗∗∗	−0.102∗∗∗	−0.100∗∗∗	−0.083∗∗∗	−0.093∗∗∗	−0.103∗∗∗	0.118∗∗∗	0.104∗∗∗	0.121∗∗∗	0.124∗∗∗
	(0.001)	(0.000)	(0.000)	(0.001)	(0.000)	(0.000)	(0.000)	(0.000)	(0.000)	(0.000)	(0.000)	(0.000)
Constant	0.279∗∗∗	0.434∗∗∗	0.335∗∗∗	0.210∗∗∗	0.426∗∗∗	0.387∗∗∗	0.279∗∗∗	0.294∗∗∗	0.301∗∗∗	0.230∗∗∗	0.274∗∗∗	0.308∗∗∗
	(0.000)	(0.000)	(0.000)	(0.000)	(0.000)	(0.000)	(0.000)	(0.000)	(0.000)	(0.000)	(0.000)	(0.000)

Note: The dependent variables are CO_2_ emissions, ecological footprint, and EPI. Coefficients are estimated using the DOLS estimator. P-values are given in parentheses. ∗, ∗∗, and ∗∗∗ indicate the significance at 10 %, 5 %, and 1 % levels, respectively. Models 1 to 3 indicate ICT use, ICT access, and ICT skills, respectively. OpE indicates opportunity entrepreneurship. (Source: authors elaboration).

**Table 9:** Robustness check: Complementarity effects of entrepreneurship and ICT on CO_2_ emissions, ecological footprint, and EPI using FMOLS estimator.

Variables	CO_2_ emissions			Ecological footprint			Environmental Performance Index (EPI)		
	ICT diffusion			ICT diffusion			ICT diffusion		
	Model 1	Model 2	Model 3	Model 1	Model 2	Model 3	Model 1	Model 2	Model 3
OpE	0.188∗∗∗	0.147∗∗	0.198∗∗∗	0.172∗∗∗	0.162∗∗∗	0.181∗∗∗	−0.102∗∗∗	−0.138∗	−0.144∗∗∗
	(0.000)	(0.018)	(0.000)	(0.000)	(0.000)	(0.000)	(0.006)	(0.056)	(0.000)
ICT use (Use)	0.077∗∗∗	–	–	0.136∗∗∗	–	–	−0.099∗∗	–	–
	(0.000)			(0.000)			(0.025)		
ICT access (Access)	–	0.130∗∗∗	–	–	0.114∗∗	–	–	−0.139∗∗∗	–
		(0.000)			(0.031)			(0.000)	
ICT skills (Skills)	–	–	0.147∗∗∗	–	–	0.105∗∗	–	–	−0.148∗∗∗
			(0.001)			(0.010)			(0.000)
Use x OpE	−0.016∗∗∗	–	–	−0.013∗∗∗	–	–	0.009	–	–
	(0.000)			(0.000)			(0.187)		
Access x OpE	–	−0.020∗∗∗	–	–	−0.017∗∗∗	–	–	0.011∗∗∗	–
		(0.000)			(0.000)			(0.000)	
Skills x OpE	–	–	−0.039∗∗∗	–	–	−0.041∗∗∗	–	–	0.009
			(0.000)			(0.000)			(0.113)
**Net effects**	**−2.173**	**−1.285**	**−0.174**	**−1.746**	**−1.055**	**−0.211**	**n.a.**	**0.650**	**n.a.**
GDP per capita	0.265∗∗∗	0.194∗∗∗	0.201∗∗∗	0.238∗∗∗	0.187∗∗∗	0.234∗∗∗	−0.212∗∗∗	−0.184∗∗∗	−0.199∗∗∗
	(0.000)	(0.000)	(0.000)	(0.000)	(0.000)	(0.000)	(0.000)	(0.000)	(0.000)
Financial development	0.148∗∗∗	0.151∗∗∗	0.137	0.143∗∗	0.149∗∗	0.169∗∗∗	−0.129	−0.131∗∗∗	−0.129∗∗
	(0.000)	(0.000)	(0.104)	(0.036)	(0.017)	(0.000)	(0.103)	(0.000)	(0.031)
Trade openness	0.324∗∗∗	0.227∗∗∗	0.195∗∗∗	0.208∗∗∗	0.188∗∗∗	0.173∗∗	−0.193∗∗∗	−0.203∗∗∗	−0.197∗∗∗
	(0.000)	(0.000)	(0.000)	(0.000)	(0.000)	(0.026)	(0.000)	(0.000)	(0.000)
Rule of law	−0.094∗∗∗	−0.091∗∗∗	−0.088∗∗∗	0.071∗∗∗	−0.106∗∗∗	−0.098∗∗	0.095∗∗∗	0.101∗∗∗	0.107∗∗∗
	(0.000)	(0.000)	(0.000)	(0.000)	(0.000)	(0.022)	(0.000)	(0.000)	(0.000)
Constant	1.123∗∗∗	1.004∗∗∗	0.944∗∗∗	0.939∗∗∗	1.089∗∗∗	0.999∗∗∗	0.789∗∗∗	0.835∗∗∗	0.849∗∗∗
	(0.000)	(0.000)	(0.000)	(0.000)	(0.000)	(0.000)	(0.000)	(0.000)	(0.000)

Note: The dependent variables are CO_2_ emissions, ecological footprint, and EPI. Coefficients are estimated using the DOLS estimator. P-values are given in parentheses. ∗, ∗∗, and ∗∗∗ indicate the significance at 10 %, 5 %, and 1 % levels, respectively. The mean values of ICT use, ICT access, and ICT skills are 147.586, 71.637, and 9.564, respectively. n.a. indicates not applicable because at least one of the coefficients used in the calculation of the net effect is not significant. Models 1 to 3 indicate ICT use, ICT access, and ICT skills, respectively. OpE indicates opportunity entrepreneurship. (Source: authors elaboration).

## 5 Conclusion and implications

Since implementing sustainable development issues in Vision 2030, policymakers have eagerly searched for practical policies and tools to foster environmental sustainability. In this pursuit, entrepreneurship and ICT have emerged as pivotal factors in achieving this ambitious objective. Therefore, the present study aims to delve into the intricate nexus between entrepreneurship, ICT, and environmental sustainability to provide valuable insights to policymakers in their quest for effective strategies and tools to promote environmental sustainability. Specifically, this article examined the linear and non-linear impacts of opportunity entrepreneurship and ICT diffusion on environmental quality, measured by CO_2_ emissions, ecological footprint, and environmental performance index. It also examined the interaction effect of ICT and opportunity entrepreneurship on improving environmental quality. This research specifically focused on Saudi Arabia, a country pivotal in the global energy market, highlighting the importance of understanding the dynamic factors that influence environmental outcomes within this context.

The findings show that opportunity entrepreneurship is associated with higher levels of environmental degradation. However, the quadratic relationship between opportunity entrepreneurship and the three proxies of environmental quality suggests that, beyond a certain threshold, increased opportunity-driven entrepreneurs could lead to improved environmental performance, supporting the notion that entrepreneurship can lead to environmental sustainability. Moreover, increased levels of ICT have been found to harm environmental quality. This negative effect is observed across ICT proxies. It can be attributed to various factors, including the energy consumption, emissions, and waste generation associated with the production, use, and disposal of ICT products. However, a quadratic relationship exists between the proxies of ICT and both CO_2_ emissions and ecological footprint, indicating that while initial increases in ICT use, access, and skills worsen environmental degradation, further increases beyond certain threshold levels contribute to improved environmental quality. In other words, there is a turning point where additional ICT usage can positively affect environmental quality. Additionally, the study's findings reveal negative net effects on both CO_2_ emissions and ecological footprint from the interactions between the proxies of ICT and opportunity entrepreneurship. In contrast, positive net effects were observed on the environmental performance index from interactions involving ICT skills and access. Specifically, higher levels of ICT were associated with enhanced environmental conditions, suggesting that ICT acts as a modulating mechanism, aligning entrepreneurial efforts with environmental sustainability goals.

This article also provides some policy and practical recommendations to improve environmental quality in line with the above findings. First, the findings underscore the complex relationship among entrepreneurship, ICT, and environmental sustainability, showing both challenges and opportunities for policymakers and stakeholders. Initially, the study highlighted that opportunity entrepreneurship tends to be associated with higher levels of environmental degradation. However, beyond a certain threshold, increased levels of opportunity entrepreneurship could potentially lead to improved environmental quality. This indicates that while entrepreneurship may initially worsen environmental degradation, a critical juncture exists where entrepreneurial effort shifts toward fostering environmental sustainability. Therefore, policymakers must recognize this threshold and develop strategies to incentivize and guide entrepreneurial activities toward more sustainable practices. This could involve providing tax incentives, grants, and technical support to opportunity entrepreneurs who prioritize eco-friendly innovations and sustainability initiatives. Moreover, the results revealed that increased levels of ICT diffusion harm environmental quality across various proxies. However, further advancements beyond certain threshold levels of ICT contribute to improved environmental quality, i.e., a turning point where additional ICT diffusion can positively affect environmental sustainability. Therefore, policymakers must consider the immediate and long-term consequences of the expanding digital technologies on environmental sustainability. During the early stages of ICT diffusion, the focus should be on promoting adoption while implementing regulations and incentives to mitigate potential negative effects. For instance, policies could encourage responsible recycling and disposal of outdated devices. As digitalization becomes more widespread in a region or industry, the emphasis should shift toward harnessing the long-term environmental benefits of ICT. Once a sufficient infrastructure and skill base are established, policies can encourage using technologies like artificial intelligence, the Internet of Things (IoT), and big data analytics to drive material reductions and efficiency gains across various sectors. Businesses can also incorporate environmental considerations into their digital transformation strategies. Initially, efforts may revolve around minimizing direct impacts through initiatives such as establishing take-back programs and utilizing renewable energy sources. As capabilities advance, the focus can shift toward leveraging emerging technologies to make existing operations, products, and services more environmentally friendly. Startups, too, can explore opportunities to develop innovative circular business models enabled by new digital tools. Finally, the study's findings highlight the crucial role of ICT diffusion in shaping the relationship between opportunity entrepreneurship and environmental quality. This emphasizes the need for policymakers to recognize the transformative potential of digital technologies in aligning entrepreneurial efforts with sustainability goals. To leverage this potential, governments should prioritize the development of comprehensive frameworks that harness ICT tools for environmental monitoring, data analysis, and decision-making processes. By integrating digital technologies into environmental governance, policymakers can enhance the effectiveness and efficiency of environmental management practices, leading to improved conservation outcomes. Promoting green ICT initiatives is also essential at both the regulatory and industry levels. Policymakers can incentivize the development and adoption of eco-friendly ICT solutions by supporting research and development efforts and implementing regulations that encourage energy-efficient ICT infrastructure and operations. Collaboration and knowledge sharing among stakeholders in the ICT and environmental sectors are also vital. Establishing partnerships between government agencies, private companies, academic institutions, and civil society organizations can facilitate the exchange of best practices, data, and resources. Through collaboration, stakeholders can leverage their collective expertise and resources to address complex environmental challenges more effectively, maximizing the positive impact of ICT on environmental sustainability.

While this study provides valuable insights into the relationship between entrepreneurship, ICT diffusion, and environmental sustainability, it is important to acknowledge certain limitations. Firstly, the research focused primarily on Saudi Arabia and can be generalized to GCC countries; however, it may restrict the generalizability of the findings to other regions with different socio-economic and environmental contexts. To enhance the applicability of the findings, future research could include a more diverse range of countries to capture the variations in this relationship across different contexts. Secondly, the study relied on proxy indicators, such as CO_2_ emissions, ecological footprint, and environmental performance index, to assess environmental quality. While these indicators offer valuable insights, they may not encompass the full environmental impacts of entrepreneurship and ICT diffusion. Future research could explore alternative measures and consider broader environmental indicators to provide a more comprehensive evaluation. Additionally, the study primarily examined the direct effects of entrepreneurship and ICT diffusion on environmental sustainability, overlooking potential mediating and moderating factors that could influence this relationship. Future research could investigate the role of factors like institutional frameworks, government policies, and cultural attitudes in shaping the environmental impacts of entrepreneurial activities and ICT adoption.

## CRediT authorship contribution statement

**Faisal Alfehaid:** Project administration, Investigation, Formal analysis, Conceptualization. **Anis Omri:** Writing – original draft, Methodology, Investigation, Formal analysis, Data curation, Conceptualization. **Ahmad Altwaijri:** Writing – original draft, Validation, Investigation, Formal analysis, Conceptualization.

## Declaration of competing interest

The authors declare the following financial interests/personal relationships which may be considered as potential competing interests:

Anis Omri reports financial support was provided by 10.13039/501100007414Qassim University, Deanship of Scientific Research. Anis Omri reports a relationship with 10.13039/501100007414Qassim University, Deanship of Scientific Research that includes: employment and funding grants. If there are other authors, they declare that they have no known competing financial interests or personal relationships that could have appeared to influence the work reported in this paper.
